# Systemic sarcoidosis presenting as acute rapidly progressive proptosis

**DOI:** 10.5935/0004-2749.20230005

**Published:** 2022-01-31

**Authors:** Maria Valeria da Silva, Yael Chavez, Maria Laura Di Nicola, Jan M. A. Delabie, Kalpana Rose, Hatem Krema

**Affiliations:** 1 Ocular Oncology Service, Princess Margaret Cancer Centre, Toronto, Ontario, Canada; 2 Department of Pathology, University Health Network, University of Toronto, Toronto, Ontario, Canada

**Keywords:** Sarcoidosis, Orbit/pathology, Exophthalmos, Lymphadenopathy, Sarcoidose, Órbita/patologia, Exoftalmia, Linfadenopatia

## Abstract

Sarcoidosis is a generalized systemic chronic inflammation that rarely involves the orbit. As a chronic inflammation, sarcoidosis typically manifests with an insidious onset and slowly progressive course. We report a case of acute-onset proptosis resulting from a rapidly growing diffuse orbital mass that simulated malignant growth, which was biopsy proven to be the first manifestation of systemic sarcoidosis. The patient demonstrated complete resolution of proptosis and systemic involvement with long-term corticosteroid treatment.

## Abstract (pt)

A sarcoidose é uma inflamação sistêmica generalizada que raramente envolve a órbita. Sendo uma inflamação crônica, a sarcoidose geralmente se manifesta com um início insidioso e uma evolução lentamente progressiva. Neste artigo, é relatado um caso de proptose de início agudo, resultante de uma massa orbitária difusa de rápido crescimento, simulando um crescimento maligno, mas cuja biópsia comprovou ser a primeira manifestação de uma sarcoidose sistêmica. O paciente apresentou resolução completa da proptose e do envolvimento sistêmico com tratamento de longo prazo com corticosteroides.

## INTRODUCTION

Orbital involvement with sarcoidosis is rare^(1)^. The ophthalmic literature tends to confuse isolated orbital granulomatous disease with sarcoidosis. Several authorities believe that orbital sarcoid should not be diagnosed in the absence of systemic disease^(2)^. We report a patient with an atypical clinical presentation of orbital sarcoidosis that was the first manifestation of a systemic disease.

## CASE REPORT

**Figure 1.**
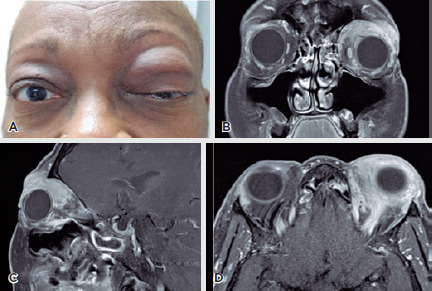
(A) Clinical picture at first presentation. A 63-year-old Afro-descendant woman presented with a 2-month history of progressive swelling of the upper left eyelid associated with painful eye movement. (B) Coronal- and (C) sagittal-, and (D) axial-view orbital magnetic resonance images showing an extensive orbital mass occupying the superior aspect of the left anterior orbit that involved the lacrimal gland, superior rectus muscle, levator muscle complex, and portion of the lateral rectus. The orbital apex was clear.

A 63-year-old Afro-descendant woman presented with a rapidly progressive large swelling and fullness of the upper left eyelid and anterior orbit that evolved within 6 weeks. Proptosis was associated with limited painful eye movements. Her visual acuity was 20/20 in the right eye and 20/30 in the left eye. The intraocular pressure was 12 mmHg in the right eye and 18 mmHg in the left. External examination of the left eye revealed a mechanical ptosis occurring secondary to a large mass involving the superior anterior orbit (Figure 1A). On palpation, it was firm, ill-defined, and non-tender, with no redness or edema in the overlying skin. On forcible opening of the eyelids, moderate proptosis with inferior displacement of the left eye was detected. The exophthalmometry measurements were 23 mm in the right eye and 27 mm in the left eye. The eye motility was completely full in the right eye, but significant vertical and mild horizontal limitations were observed in the left eye. The result of the bilateral ocular examination was otherwise normal.

The patient consented to the publication of this case report and its related images.

### Differential diagnosis

On the basis of the absence of signs of acute inflammation and the rapidly progressive course, the provisional differential diagnosis included an acute lymphoproliferative process or a malignant infiltrative neoplasm of either primary or metastatic origin.

### Investigations

Magnetic resonance imaging revealed an extensive orbital mass occupying the superior aspect of the left anterior orbit that involved the lacrimal gland, superior rectus muscle, levator muscle complex, and part of the lateral rectus. The orbital apex was clear (Figure 1 B,C,D).

**Figure 2.**
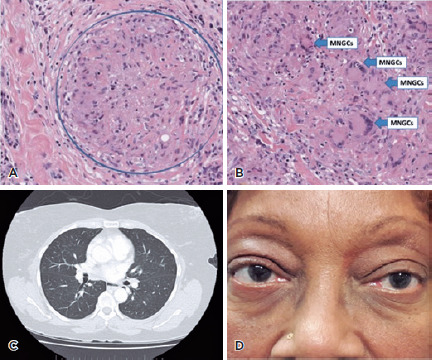
Histopathology of an incisional biopsy showing (A) many “naked” non-necrotizing granulomas. (B) As shown, the stroma was composed of epithelioid cells, scattered small lymphocytes, histiocytes, and numerous multinucleated giant cells (MNGCs). (C) The thoracic computed tomography scan shows prominent and enlarged mediastinal and bilateral hilar lymph nodes with no parenchymal manifestation. (D) The clinical picture of the patient after 2 years of diagnosis without signs of relapse of the disease in the orbit.

Histopathological examination of an incisional biopsy revealed many “naked” non-necrotizing granulomas (Figure 2A). The stroma was composed of epithelioid cells, scattered small lymphocytes, histiocytes, and numerous multinucleated giant cells (Figure 2B). Special staining for acid-fast bacilli and fungal organisms did not reveal any microorganisms. The non-necrotizing granulomatous inflammation was most consistent with sarcoidosis. Consequently, the patient submitted to systemic screening for sarcoidosis. On thoracic computed tomography (CT), prominent and enlarged mediastinal and bilateral hilar lymph nodes with no parenchymal manifestations were detected (Figure 2C).

### Treatment

The patient was treated initially with 50 mg of prednisone, and the dose was tapered slowly over a few months to a maintenance dose of 5 mg every other day.

### Outcome and follow-up

The patient demonstrated rapid regression of proptosis and ptosis with full restoration of ocular motility. The CT scan performed 2 months after the treatment revealed a significant interval decrease in the size of the infiltrative soft tissue mass in the left orbit, ruling out the provisional differential diagnosis. No signs of relapse of the disease were found in the orbit or thorax 4 years after diagnosis (Figure 2D).

## DISCUSSION

Sarcoidosis is a generalized chronic multi-systemic inflammation. It has a predilection for females in their fifth to seventh decades of life and patients of African descent^(3)^. Ophthalmic involvement occurs in almost 25% of patients with sarcoidosis, manifesting mostly as chronic uveitis, with orbital involvement representing <1% of the ophthalmic manifestations^(1^, ^4)^. However, orbital involvement frequently occurs without prior history of systemic sarcoidosis^(1)^.

In a large series of orbital lesions, it represented <0.2% of all lesions and 2% of the orbital inflammatory lesions^(5)^. Our patient has the typical demographics of a patient with sarcoidosis; however, the acute rapidly progressive course of few weeks for such an extensive orbital lesion is atypical for a chronic inflammatory process. In a series of orbital and adnexal sarcoidosis, the most common complaint was a slowly progressive mass in 88.5% of the cases, with discomfort in 30%^(1)^. The rapidly progressive clinical presentation in our patient was suggestive of a malignant process, which was refuted by the orbital biopsy findings, presence mediastinal lymph node enlargement in the subsequent thoracic imaging, and the prompt response to steroid treatment. Albeit rare, our case emphasizes the importance of including sarcoidosis in the differential diagnosis of acute rapidly progressive orbital lesions and considering systemic surveillance of the disease if biopsy revealed orbital sarcoidosis.
